# Characterizing Participants in the ClinSeq Genome Sequencing Cohort as Early Adopters of a New Health Technology

**DOI:** 10.1371/journal.pone.0132690

**Published:** 2015-07-17

**Authors:** Katie L. Lewis, Paul K. J. Han, Gillian W. Hooker, William M. P. Klein, Leslie G. Biesecker, Barbara B. Biesecker

**Affiliations:** 1 Medical Genomics and Metabolic Genetics Branch, National Human Genome Research Institute, Bethesda, Maryland, United States of America; 2 Maine Medical Center Research Institute, Center for Outcomes Research and Evaluation, Scarborough, Maine, United States of America; 3 Social and Behavioral Research Branch, National Human Genome Research Institute, Bethesda, Maryland, United States of America; 4 NextGxDx, Franklin, Tennessee, United States of America; 5 Division of Cancer Control and Population Sciences, National Cancer Institute, National Institutes of Health, Bethesda, Maryland, United States of America; South Texas Veterans Health Care System and University Health Science Center San Antonio, UNITED STATES

## Abstract

Genome sequencing is a novel clinical tool that has the potential to identify genetic origins of disease. However, the complexities of this new technology are significant and little is known about its integration into clinical care, and its potential adoption by patients. Expectations of its promise for personalized medicine are high and it is important to properly match expectations to the realities of the test. The NIH ClinSeq cohort study pilots the integration of genome sequencing into clinical research and care to assess the technical, medical and socio-behavioral aspects of implementing this technology. Over 950 adults ages 45-65 have been enrolled and clinically phenotyped. As an initial study, we describe the personality traits of ClinSeq participants, and explore how these traits compare to those that characterize early adopters of other new technologies. Our analysis was conducted on responses from 630 members of the cohort who completed a baseline survey on health cognitions, affect, health-related behaviors and personality traits, prior to receipt of any genome sequencing results. The majority of participants were white (90.5%), had at least a college degree (86.5%), and had at least one biological child (74.6%). Members of this ClinSeq sample were found to be high in dispositional optimism and resilience. Their high SES paralleled that of other early adopters of new technology. These attributes may contribute to participants’ expectations for favorable outcomes and willingness to take higher risks when compared to the general population. These characteristics may distinguish those who are most likely to pursue genome sequencing and be indicative of their psychological resources to manage returned results.

## Introduction

Genome sequencing is a new technology that allows for the sequencing and analysis of the majority of an individual’s genes simultaneously [1]. It differs from previous genetic testing paradigms in the breadth of information it can provide and the possibility to detect secondary or incidental findings unrelated to the primary reason for doing the testing [2–3]. The cost of genome sequencing continues to decline steadily and it is becoming feasible to offer the testing to a growing number of patients and research participants. As its use widens, there is a growing need to characterize the behavioral attributes of individuals who utilize genome sequencing, which may be useful in pretest counseling to set appropriate expectations and to prepare patients for the potential receipt of a variety of test results including secondary findings, and findings of uncertain significance.

ClinSeq was designed to pilot integration of genome sequencing into clinical settings [2]. The phenotype of initial interest was coronary artery disease (CAD) based on its prevalence and potential to serve as a model for the study of the genetic architecture of common disease. Participants with a spectrum of CAD risk were recruited and received an initial evaluation that focused largely on cardiovascular health. To ensure diversity in their CAD phenotype, enrollees were divided into “bins” based on their Framingham risk score (bin 1 = <5% 10-year-risk for the development of coronary artery disease, bin 2 = 5–10% risk, bin 3 = >10% risk, and bin 4 = known CAD), with a target of 25% of all participants having CAD. Although the project initially focused on CAD, participants were broadly consented for iterative evaluation of all heritable traits through genomic interrogation [4]. Participants were also consented to have the opportunity to learn their clinically relevant sequence results as they became available. This longitudinal design enables the investigation of long-term outcomes, such as psychological responses to the return of results and implementation of recommendations to purse health-related behaviors.

By definition ClinSeq participants are early adopters. They volunteered to participate in our NIH sequencing protocol as early as 2007, and data from a subset of them indicated that they were motivated by their interest in science, the new technology and what they could learn by way of results, as well as expressing an interest in furthering research [5]. That publication characterized the ClinSeq population as similar to “early adopters” of new technologies based on their sociodemographic attributes and motivations. We therefore have evidence that they are early adopters. Other studies have shown that early adopters of direct-to-consumer genetic testing are more likely to be older, white, and highly educated [6–8]. According to the diffusion of innovations theory [9], early adopters are amongst the first individuals to implement a new idea, technology or practice within their community, and are more likely to have elevated social status, high income, and higher education than late adopters. Early adopters are often people who have leadership positions within their community, and they typically play a role in spreading the use of new technology through their communication with others.

Previous studies from the business sector have attempted to characterize the personality traits of early adopters in order to better describe this group and distinguish them from groups of later adopters. In theory, early adopters should be more tolerant of risk and uncertainty (more comfortable with uncertainty across life’s experiences [10]), higher in dispositional optimism, more open to new experiences, and more resilient in the face of adverse circumstances. There is empirical support for these predictions. Early adopters consistently score higher on the openness to experience subscale on personality inventories, although evidence on the association of early adoption and conscientiousness or extraversion is mixed [11–13]. Georgsdottir and Getz [14] hypothesized that openness to experience may be correlated with the adoption of new technology because it captures an individual’s flexibility, which is a key antecedent of innovation. Those who are more tolerant of uncertainty are also more apt to use novel technologies, accepting their unknowns. Other studies have shown that dispositional optimism is also common among early adopters of technology [15–16].

No similar data exist, however, regarding the personality characteristics of early adopters of genome sequencing technology. This is an important knowledge gap, given that these personality characteristics may predispose people towards particular psychological and behavioral responses to genome sequencing information. For example, high dispositional optimism might bias people towards unrealistic expectations of benefit from sequencing or excessive confidence in sequencing results. On the other hand, resilience may protect people against negative outcomes from the return sequencing results, such as excessive worry or distress. Understanding the attributes of early adopters of genome technology will be useful in helping clinicians to anticipate people’s responses to sequence results, and to contrast this group with later adopters. This understanding may someday allow clinicians and researchers who offer genome sequencing to patients and subjects to more effectively identify individuals who are likely to cope well with their results, and to develop interventions aimed at enhancing coping for those who may be at risk for poorer outcomes.

## Materials and Methods

### Participants

The participants for this analysis were recruited from the larger ClinSeq study. Eligible participants were told that the study would pilot the use of genome sequencing and they would have the opportunity to learn results. Approximately one quarter of the participants had a personal history of CAD, however the majority of ClinSeq participants were not selected for any health problems (described here as “healthy volunteers”). The healthy volunteers were recruited from the general population through advertisements in newspapers, public transit, and other public spaces in the greater Washington DC area. The participants with CAD were recruited through similar venues as the healthy volunteers, as well as targeted recruitment through a local cardiac rehabilitation program in which a nurse approached eligible participants with a brochure and an offer to pass their contact information to the ClinSeq study team if they were interested in participating. Individuals were then screened to determine whether they met various eligibility criteria, including falling between the ages of 45–65. Individuals under the age of 45 were not enrolled in because they were less likely to have coronary artery disease, an initial risk of interest to the investigators. Individuals over the age of 65 were not enrolled in order to allow for the study of long-term outcomes, given the longitudinal nature of the study.

At the time of this analysis, over 950 participants were enrolled in ClinSeq. All participants were broadly consented to genome sequencing in a research setting, including return of genetic results, and re-contact for additional phenotyping or ancillary study enrollment. Participants were not compensated for their involvement. Each participant also provided self-reported demographic data including: age, gender, education, income, number of children, marital status, race, and ethnicity. Several of the demographic variables were dichotomized prior to analysis including: income (>$100,000 per year vs. ≤ $100,00 per year), education (completed college or more vs. some college or less), CAD status (not affected vs. affected), and number of children (1+ vs 0).

### Data Analysis

Additional data for these analyses were collected as part of a survey on health-related socio-behavioral factors. ClinSeq participants who were enrolled for at least one month but had not yet received a sequence result were eligible to complete the survey. Participants were recruited by mail, phone, or secure email. The survey was available both online and in a paper version; scales were administered in random order for each participant taking the online survey. Surveys responses were collected from August 2012 to April 2015. The survey stated that participant consent to participate was implicit in their completion of the survey, which was approved by the National Human Genome Institute (NHGRI) Institutional Review Board (IRB).

The survey included 34 scales, ranging in size from 1–45 items, and took approximately 45 minutes to complete. The relevant scales for these analyses included traits that have been previously linked to medical outcomes, such as pursuit of health-related behaviors, including:

- **Tolerance for Uncertainty**
  Tolerance for uncertainty was assessed using the 7-item Tolerance for Ambiguity scale [17]. This scale assesses the extent to which individuals are comfortable with uncertain situations. We refer to the scale as a measure of uncertainty because it does not assess “ambiguity” as defined in the literature [18–19]. Items were scored on a scale from 1 (Not at all characteristic of me) to 5 (Entirely characteristic of me), and were summed and averaged for a total score. Higher scores represent **less** tolerance for uncertainty. The α coefficient was 0.80.
- **Optimism**
  Optimism refers to an individual’s ability to view various life experiences and circumstances positively. We assessed dispositional optimism using the 3-item optimism subscale from the Life Orientation Test—Revised (LOT-R) [20]. Each item was rated on a 5-item scale. A total optimism score was obtained by summing the responses with higher scores representing greater optimism. The α coefficient was 0.845.
- **Personality Traits**
  Personality traits were assessed using a version of the Big Five Inventory [21], a 44-item measure of five dimensions of personality including: extraversion, agreeableness, conscientiousness, neuroticism, and openness to experience. Extraversion includes being sociable, energetic and outgoing; agreeableness includes warmth, sympathy, and compliance; conscientiousness refers to efficiency, organization and thoroughness; neuroticism refers to irritability, shyness and vulnerability; and openness to experience includes curiosity, wide-ranging interests and excitability. Each item was rated on a 5-point scale from 1 (disagree strongly) to 5 (agree strongly). Overall scores were calculated as an average of the items representing each dimension, some of which are reversed scored [21]. Alpha coefficients for each scale were 0.843 (neuroticism), 0.668 (agreeableness), 0.814 (conscientiousness), 0.824 (openness), and 0.795 (extraversion).
- **Resilience**
  Resilience refers to the ability to recover in the face of difficulty and was assessed using the RS-14 [22]. Respondents were asked to rate their agreement with 14 statements on a scale from 1 (Strongly Disagree) to 7 (Strongly Agree). A total resilience score was calculated from the sum of all items. This scale has previously been used in a variety of populations and has good internal consistency (α = 0.93).

Data were imputed for missing items on each scale when the participant completed the majority of items on the scale; the average of the individual’s responses to the other scale items was used as the imputed value, after reverse scoring specific items. On average, this occurred within one scale for 14% of respondents. If a participant did not complete more than half of the scale, their responses to that measure were not included in the analyses. Data were analyzed using SAS software (SAS Institute, Cary, NC).

### Ethics Statement

This research was reviewed and approved by the National Human Genome Research Institute (NHGRI) Institutional Review Board (IRB) at the National Institutes of Health. Participants signed a written informed consent document for the overall protocol at the time of enrollment. The survey stated that participant consent to participate was implicit in their completion of the survey, which was approved by the National Human Genome Institute (NHGRI) Institutional Review Board (IRB). Survey data were also de-identified prior to analysis.

## Results

Survey responses were received from 674 of 969 eligible participants. Eight potential participants declined to complete the survey, and the other 296 subjects were deemed “passive refusals” after failing to respond to three contact attempts. Seventy-four of the surveys were completed on paper and 600 were completed electronically. After eliminating surveys that had missing responses for more than half of all survey items (N = 43), or missing responses for all of the measures included in these analyses (N = 1), a total of 630 unique survey responses remained for analysis, for an overall response rate of 65%.

The majority of survey respondents were non-Hispanic whites (90.5%), college graduates or beyond (86.5%), not affected with coronary artery disease (79.5%), the parent of at least one biological child (74.6%), earning a household income over $100,000 per year (74.1%), and married (74.6%) (see Table 1). The ratio of male to female respondents approached 1.0. On average, participants were 61 years old (± 5.5 years). These data mirror previous descriptions of the ClinSeq cohort as highly educated, white, non-Hispanic or Latino participants [2,5]. The demographics of the survey respondents are comparable to those reported in other studies of populations seeking out genetic testing, many of which have reported racial disparities [23–25]. Similarly, early users of direct-to-consumer genetic testing are reportedly in their late 40’s [6] or 50’s [7]. While our population is not demographically representative of the U.S. population, it is likely similar to those who will seek genome sequencing in the near term.

**Table 1:** Demographics of Respondents versus All Participants.

	Survey Respondents		Survey Non-Respondents		Χ^2^ statistic and p value
	N (Total = 630)	%	N (Total = 339)	%	
Ethnicity/Race					Χ^2^ = 43.2, p < 0.01
Not Hispanic or Latino/White	570	90.5%	253	74.6%	
Other/Unknown/Not Reported	60	9.5%	86	25.4%	
Time from Consent to Survey (or survey midpoint)					Χ^2^ = 102.3, p <0.01
<1	39	6.2%	10	2.9%	
1–2	42	6.7%	14	4.1%	
2–3	60	9.5%	21	6.2%	
3–4	135	21.4%	30	8.8%	
4–5	194	30.8%	72	21.6%	
5–6	120	19.0%	123	36.3%	
6+	40	6.3%	69	20.1%	
Age (years) (at time of survey or midpoint)					Χ^2^ = 7.8, p = 0.17
<50	22	3.5%	12	3.5%	
50–54	80	12.7%	53	15.6%	
55–59	138	21.9%	75	22.1%	
60–64	203	32.2%	98	28.9%	
65–69	170	27.0%	82	24.2%	
70+	17	2.7%	19	5.6%	
Bin					Χ^2^ = 0.15, p = 0.70
1–3	501	79.5%	266	78.5%	
4	129	20.5%	73	21.5%	
Education					Χ^2^ = 21.3, p<0.01
College Graduate and Higher	545	86.5%	255	75.2%	
Less than College Graduate	66	10.5%	58	17.1%	
Not collected/Not reported	19	3.0%	26	7.7%	
Income					Χ^2^ = 20.2, p <0.01
More than $100,000	467	74.1%	210	61.9%	
$100,000 or less	132	21.0%	91	26.8%	
Not collected/Not Reported	31	4.9%	38	11.2%	
Sex					Χ^2^ = 3.94, p = 0.04
Male	345	54.8%	163	48.1%	
Female	285	45.2%%	176	51.9%	
Marital Status					Χ^2^ = 1.17, p = 0.28
Married	470	74.6%	242	71.4%	
Other/Unknown	160	25.4%	97	28.6%	
Number of Children					
1+	470	74.6%			
0	160	25.4%			

Survey respondents were significantly more likely to be non-Hispanic whites, more recently consented to the study, college graduates or higher, and men, and to have an income >$100,000 per year than members of the overall ClinSeq population.

### Measures

#### Tolerance for Uncertainty

Tolerance for uncertainty was normally distributed with a mean of 2.6 out of 5 (5 indicates the lowest degree of tolerance) and a SD of 0.78. The population was not high in tolerance for uncertainty as hypothesized.

#### Optimism

As has been previously reported [26], the average score on the optimism scale was 8.32 on a scale of 0–12 (SD: 2.2), indicating respondents have high levels of dispositional optimism.

#### Big Five Personality Traits

The scores for each scale are reported in Table 2. Survey respondents rated themselves lowest on neuroticism, and highest on agreeableness, openness, and conscientiousness.

**Table 2:** Big Five Scores.

	Average	St Dev	Range	Cronbach’s alpha
Extraversion (N = 604)	3.34	0.72	1–5	0.795
Neuroticism (N = 604)	2.42	0.76	1–5	0.843
Conscientiousness (N = 604)	4.04	0.60	1–5	0.814
Openness (N = 603)	3.90	0.61	1–5	0.824
Agreeableness (N = 604)	3.95	0.50	1–5	0.668

#### Resilience

The average resilience of survey respondents was 85.8 on a scale from 14–98, with a standard deviation of 10.8, indicating a high level of resilience.

## Discussion

This analysis provides a more comprehensive description of the ClinSeq cohort than has previously been published. The demographic data from this survey support previous descriptions of the population as early adopters with high education and income levels [5], [27]. The high levels of formal education and knowledge of the participants regarding genome sequencing [28] likely predispose them to engage in clinical research. Although the demographic characteristics of this group indicate that they are not representative of the general U.S. population, they are similar to early adopters of direct-to-consumer genetic testing [6–8] and likely similar to those who will seek genome sequencing in the near term. These results provide a characterization of early adopters, who will be the recipients of genome sequencing for the foreseeable future. Their demographics and personality traits may help researchers and clinicians anticipate whether the traits that characterize them, such as optimism and resilience, also influence their responses to the technology. For example, personality traits can predict or moderate uptake and use of health information, as has been shown in a number of health care settings [29]. Three recent hypothesis-generating studies demonstrate that personality traits and psychological factors affect intentions to learn results from genome sequencing [26,30–31]. Dispositional optimism is an example of a trait that affects health-related choices. In one early analysis, we found that greater optimism and perceived risk interacted to predict intentions to learn results from sequencing. In addition, these early adopters are an important group to characterize because they will play a key role in shaping future use of this technology [32] and recruiting subsequent waves of adopters [9]. The experiences of early adopters can help investigators returning results anticipate factors that may affect decisions by downstream users. Outcomes for early adopters are likely to be more favorable than for downstream users due to their cognitive and affective resources, access to services and personality traits. As such, any struggles or obstacles observed may suggest that there will be similar and greater challenges among a broader population with fewer resources. The current study provides important baseline data for comparing, contrasting, and better understanding the psychological attributes of future genome sequencing recipients.

Similar to other populations of early adopters, ClinSeq participants were high in dispositional optimism [15–16]. Optimists cope differently with stressful situations than pessimists because the former believe that positive outcomes are attainable. Optimism also predicts lower levels of clinical anxiety in response to receipt of positive genetic testing results [33], which may make the potential psychological and emotional risks of receiving individual genetic testing results less distressing for these participants. This may ultimately mean that these participants are more receptive to learning results from genome sequencing. At the same time, the observed high resilience of the cohort—which protects people from adverse effects that may emanate from receiving testing results—is also important to how early adopters may respond to their results. A previous literature review on the impact of receiving genetic testing results found that although testing results caused short-term distress, there were no significant long-term adverse psychological effects [34], and suggested that there may be a selection bias amongst the individuals who seek genetic testing. This provides support from another genetic testing context for our thesis that early adopters may have personality traits that not only influence their proclivity for early adoption, but which also have bearing on outcomes of their receipt of results. Those individuals who elect to have genetic testing may have more resources to cope effectively with their results. The resilience observed in the ClinSeq population may reflect a similar selection bias. However, the high optimism and resilience of the current ClinSeq cohort suggest that these early adopters of genome sequencing are less likely to experience negative psychological outcomes upon receipt of results. This is supported by the broaden-and-build theory of positive emotions, which suggests that when an individual responds to a situation with positive affective resources, such as optimism or resilience, the range of possible thoughts and actions broadens, which in turn, helps regulate negative affect [35].

Participants were also high in agreeableness, openness and conscientiousness, resembling the pattern observed in a cohort of 162 60-year-old American Internet users [36]. Individuals who are high in both conscientiousness and agreeableness have been previously described as “effective altruists” because of their motivation to not only achieve personal goals, but also work toward the greater good of a group [37]. This characterization is consistent with the self-reported motivations of ClinSeq research participants, who joined the study not only with the hopes of gaining personal benefit, but also for altruistic reasons [5]. This may provide evidence that these participants, and possibly other early adopters, will continue with participation in order to benefit the greater good even if their results are unexpected or disappointing. Although studies have found that individuals who are high in conscientiousness are less likely to engage in negative health behaviors, such as excessive drinking or smoking tobacco [38–39], one recent study found that conscientiousness was associated with the greatest health benefits when it was coupled with high agreeableness [40]. However, a previous study of over 2,000 early adopters of DTC genetic testing found that testing results and magnitude of risk did not lead to changes in health behaviors, such as dietary fat intake or exercise frequency [41]. Overall, social and behavioral studies suggest that health behavior change is motivated by a complex interaction of factors and is unlikely to be predicted by personality traits alone. Yet, the combination of high levels of formal education and genetic knowledge, strong motivations to learn results, optimism, resilience, conscientiousness and agreeableness may represent a profile of early adopters that is associated with improved health outcomes. Research is needed to determine whether these early adopters may be more likely to follow through on recommended behavior changes or seek out additional information on their individual genomic results.

Survey response and completion rates in this sample exceeded our expectations, given that no incentive was offered and it took an average of 45–50 minutes to complete. In this case, the response rate also suggests that participants are willing to continue to invest time and effort into the longitudinal cohort study. The high response rate is also consistent with earlier data suggesting these participants are highly motivated not only by the goal of bettering their personal health, but by altruism and a desire to move the science forward [5].

This paper accomplishes an important objective in characterizing the population of ClinSeq participants using baseline survey data. There are a number of studies underway that are using data from this cohort and it is key that collaborators describe the population similarly. In publishing the description, subsequent publications can refer to it to standardize descriptions of the population.

Understanding psychological factors that facilitate prolonged engagement with subjects in a genetic sequencing cohort study is critical for iterative phenotyping [4] and asking multiple research questions that require longitudinal data. These questions include whether attitudes and expectations correlate with adoption of screening recommendations and changes to medications, or with participant preferences for the types of results they are offered, or the method of result delivery. Longitudinal research on such outcomes of disclosing genomic information is of critical importance given the nascent status of clinical genomics and the unique nature of these results in both breadth and inherent uncertainty, which may impact outcomes of result disclosure, such as communication of results and emotional response of the individual participant [42–44]. Understanding these relationships will offer insights on the degree to which disclosure needs to be tailored to each individual participant, which is a key factor in determining the burden of result disclosure. Furthermore, research has provided evidence that attitudes toward genome sequencing results are likely newly-formed and highly susceptible to change [45–46], making it critical to assess them over time and in reaction to actual results.

This study is limited by the self-selection of respondents. The ClinSeq cohort is a self-referred cohort of participants who have sought out genome sequencing and may, therefore, be different from those who are offered testing in clinical and other research settings. While these factors may limit the generalizability of results to a broad population, the demographics of this sample are similar to those in other individuals undergoing genetic testing. Survey respondents were also more likely than non-respondents to be white, male, higher in education and higher in income, which may limit the applicability of these findings to the full ClinSeq population.

Future studies are needed to better understand how to learn from this population’s attributes to predict relevant outcomes of genetic sequencing in other groups, including those who are not early adopters. Downstream studies will be key to fully characterizing the broader population of those who come use sequencing to improve their health. It will also be important to study other outcomes and attributes of the present cohort such as health behaviors or communication. The ClinSeq cohort provides a rich resource for conducting such studies in the future, and the current initial study endorses the value of this work. Further analyses will be conducted to learn more about how various social and behavioral characteristics are related to key behavioral outcomes in this context.

## Supporting Information

S1 Table Individual Survey Response Data. (XLS)
